# miR-146a-mediated suppression of the inflammatory response in human adipocytes

**DOI:** 10.1038/srep38339

**Published:** 2016-12-06

**Authors:** Julian Roos, Eveliina Enlund, Jan-Bernd Funcke, Daniel Tews, Karlheinz Holzmann, Klaus-Michael Debatin, Martin Wabitsch, Pamela Fischer-Posovszky

**Affiliations:** 1 Division of Pediatric Endocrinology and Diabetes, Department of Pediatric and Adolescent Medicine, University Medical Center Ulm, Ulm, Germany; 2 Core Facility Genomics, Ulm University, Ulm, Germany; 3 Department of Pediatric and Adolescent Medicine, University Medical Center Ulm, Ulm, Germany

## Abstract

The obesity-associated inflammation of white adipose tissue (WAT) is one of the factors leading to the development of related diseases such as insulin resistance and liver steatosis. Recently, microRNAs (miRNAs) were identified as important regulators of WAT functions. Herein, we cultured human Simpson-Golabi-Behmel syndrome (SGBS) adipocytes with macrophage-conditioned medium (MacCM) and performed an Affimetrix miRNA array to identify miRNAs differentially expressed under inflammatory conditions. We identified 24 miRNAs differentially expressed upon inflammation in human adipocytes and miR-146a was the most up-regulated miRNA species. In subcutaneous WAT, miR-146a was elevated in both human and murine obesity. Transfection of miR-146a mimics prevented the MacCM-induced inflammatory response in SGBS adipocytes as seen by reduced levels of IL-8 and MCP-1 mRNA and protein. We identified IRAK1 and TRAF6 as targets of miR-146a in human adipocytes and detected a reduced inflammation-induced activation of JNK and p38 upon miR-146a transfection. Taken together, we could show that miR-146a reduces the inflammatory response in human adipocytes. In a negative feedback loop miR-146a might contribute to the regulation of inflammatory processes in WAT and possibly prevent an overwhelming inflammatory response.

The increasing prevalence of obesity and its related co-morbidities stimulated the research on adipose tissue biology. Several studies in the last years revealed that white adipose tissue (WAT) is not a simple energy storage organ, but influences the whole organism by the secretion of adipokines, which are involved in important functions including metabolism, energy homeostasis and inflammation^1^. In the obese state, the secretome of adipocytes and adipose tissue as a whole shifts from predominantly anti-inflammatory to mainly pro-inflammatory, causing a local low-grade inflammation^2^. Many of the secreted factors, *e.g*. monocyte chemotactic protein 1 (MCP-1), tumor necrosis factor α (TNF-α), interleukin (IL)-1, IL-6 and IL-8 promote severe co-morbidities including type 2 diabetes mellitus, liver steatosis and cardiovascular diseases including hypertension and atherosclerosis^3^,^4^,^5^,^6^. Classical interventions against obesity such as low caloric diet and physical exercise usually fail in the long run, while bariatric surgery results in sustainable weight reduction and improvements of metabolic parameters in most cases, yet carries inherent risks. Anti-obesity drugs still lack physiological specificity and cause enormous adverse side effects^7^. It is therefore still indispensable to improve our knowledge on the molecular mechanisms of obesity to generate novel biomarkers to identify those patients who are at special risk to develop obesity-associated disorders and to develop new, sustainable therapeutic strategies.

microRNAs (miRNA) represent highly promising candidates in this respect^8^. miRNAs are a class of small, non-coding RNA molecules, only 18–25 nucleotides in length^9^. They play an important role in the regulation of cellular gene expression by either suppressing translation of protein coding genes or by cleaving target mRNA to induce their degradation^8^,^9^. Around 300 conserved miRNAs are encoded by the mammalian genome^8^. They regulate a plethora of biological processes, including development, differentiation, and metabolism^8^. Numerous studies have demonstrated that miRNAs are not only found intracellularly, but also detectable outside cells, including various body fluids such as serum, plasma, saliva, urine, and breast milk^10^. In human WAT, 155 miRNAs were found and their impact ranges from regulating adipogenesis and glucose metabolism to the modulation of inflammation^11^,^12^. Only recently, evidence of miRNA dysregulation was reported in human obesity and type 2 diabetes mellitus^12^,^13^,^14^.

miRNA-146a, which is encoded on human chromosome 5q33.3^15^, is known to play a critical role in immune responses in mice^16^,^17^,^18^. The knowledge on miR-146a function in the human context is limited, but some studies confirm its regulatory role in the inflammatory response akin to what has been found in mice^19^,^20^,^21^. Interestingly, a clinical study recently reported a significant reduction of miR-146a and increased pro-inflammatory IL-8 in serum of type 2 diabetes patients compared to a non-diabetic control group^22^.

The function of miR-146a in human adipocytes is currently unknown. The aim of this study was to elucidate a possible relationship between miR-146a and the inflammation of the WAT.

## Materials and Methods

If not otherwise stated, cell culture material was obtained from Sarstedt (Germany) and standard chemicals from Sigma-Aldrich (Germany). Cell culture media, buffers, and supplements were obtained from Life Technologies (Germany).

### Ethical Note

All procedures involving human subjects were approved by the ethics committee of the University of Ulm (entry number 368/13). Written informed consent was obtained from all subjects and all associated methods were conducted in accordance with approved guidelines for human experimental research.

All animal studies were approved by the local authorities (Regierungspräsidium Tübingen, entry number 1154) and experiments were performed in accordance with Germany’s laws and the rules and regulations governing animal research in the European Union.

### Study subjects

Mammary WAT samples were obtained from 21 female subjects undergoing plastic surgery. Stromal-vascular cells were isolated from three subjects by collagenase digestion and cultured according to established protocols^23^. For whole adipose tissue RNA isolation, a small piece of tissue from 18 subjects was snap-frozen in liquid nitrogen and stored frozen until isolation.

### Cell culture

All cell culture experiments were performed in at least three independent experiments. Simpson-Golabi-Behmel syndrome (SGBS) preadipocytes and primary preadipocytes were cultured as described^24^. Adipogenic differentiation was induced in sub-confluent cultures as described previously^25^. In brief, cells were washed once with PBS and serum-free DMEM-F12 supplemented with 10 μg/ml transferrin, 20 nM insulin, 100 nM cortisol, 200 pM T3, 25 nM dexamethasone, 250 μM IBMX and 2 μM rosiglitazone was added. After 4 days, medium was changed and serum-free DMEM-F12 supplemented with 10 μg/ml transferrin, 20 nM insulin, 100 nM cortisol, 200 pM T3 was added. Cells were used for experiments on day 14 of adipogenesis.

As model of inflamed adipose tissue, SGBS adipocytes were stimulated with macrophage-conditioned medium (MacCM) following a previously established protocol^26^. In brief, human THP-1 monocytes were stimulated with 125 ng/ml PMA in order to differentiate them into macrophages. Conditioned media were collected from 1 million cells/ml incubated in RPMI medium supplemented with 0.1% BSA for 48 h. Control medium was RPMI with 0.1% BSA, which was incubated for 48 h without cells. Adipocytes were treated with 10% MacCM or 10% of control medium.

### Animal experiments

Six week old male C57B/6JRj mice received either standard purified mouse diet (chow, D12450 mod. LS 13% kJ fat) or a high fat diet (EF D12492 (I) mod. 60% kJ fat). Diets were obtained from Ssniff (Spezialdiäten, Germany). After 8 weeks mice were weighed, sacrificed and subcutaneous, inguinal adipose tissue was dissected and weighed. Adipose tissue was kept in RNAlater (Thermo Fisher Scientific, USA) solution and stored at −80 °C until isolation of RNA.

### Array

One μg of total RNA was labeled using the FlashTag Biotin HSR labeling Kit (Genisphere, USA). 21.5 μl biotin-labeled sample were hybridized to GeneChip miRNA Arrays at 48 °C and 60 rpm for 16 h. After washing at a Fluidics Station 450 using fluidics script FS450_0003, arrays were scanned with an Affymetrix GeneChip Scanner 3000. Raw data were background corrected and quantile normalized using the miRNAQC Tool from Affymetrix with default parameters recommended by Affymetrix.

A miRNA transcriptome analyses was performed using BRB-ArrayTools developed by Dr. Richard Simon and BRB-ArrayTools Development Team (http://linus.nci.nih.gov/BRB-ArrayTools.html) using only miRNAs which were present in at least 50 percent of the samples.

Class Comparison: We identified genes/miRNAs that were differentially expressed among the two classes using a 2 sample t-test. Genes were considered statistically significant if their p value was <0.05 and displayed a fold change between the two groups of at least 1.5 fold.

### Transfection of miRNA mimics

SGBS adipocytes or human primary *ex vivo* differentiated adipocytes were transfected with 20 nM miR-146a mimic (Syn-hsa-miR-146a-5p, Qiagen, Germany) or non-target control (AllStars Negative Control siRNA, Qiagen, Germany) and 0.66 μl/cm^2^ Lipofectamine 2000 (Invitrogen, Germany) according to the manufacturer’s protocol. Lipofectamine/RNA complexes were added drop wise to the cells without a change of media. Transfection was validated after 48 h by assessing the levels of the transfected miRNA mimic by qPCR.

### RNA isolation

Total RNA isolation was performed with the Direct-zol RNA mini Prep Kit (Zymo Research, Germany). After rinsing once with PBS, cells were harvested with Tri-Reagent and stored at −80 °C until RNA isolation. After finishing an experiment, all of the collected samples were thawed at once and total RNA, including miRNA, was purified according to the manufacturer’s protocol. Whole adipose tissue samples were thawed on ice and then homogenized in TRI Reagent using tissue homogenizing CKMix tubes (Bertin Technologies, France) and a TissueLyser LT (Qiagen, Germany) homogenizer. Chloroform (Carl Roth, Germany) was added to 20%v/v, the homogenates were shaken vigorously and centrifuged at high speed. The upper, colorless phase was separated and used for actual isolation.

### Reverse transcription and quantitative PCR (qPCR)

For miRNA quantification, total RNA was reverse transcribed using the miScript II RT Kit (Qiagen, Germany) and analysed by real-time qPCR using the miScript SYBR Green PCR Kit and the miScript primer assay for Hs-miR-146a_1 (Qiagen, Germany). Results were normalized to SNORD68_11 (sno68) (Qiagen, Germany) using the 2^−ΔCt^ or 2^−ΔΔCt^ method^27^.

To investigate mRNA expression, total RNA was reverse transcribed using SuperScript II Reverse Transcriptase (Invitrogen, Germany). qPCRs were performed with the My Budget 5x EvaGreen qPCR-Mix (Bio Budget, Germany) and the primers given below. qPCRs for IRAK1 were performed with LightCycler Fast Start DNA Master SYBR Green I (Roche, Germany). Results were normalized to SDHA or HPRT using the 2^−ΔCt^ or 2^−ΔΔCt^ method^27^. All qPCR experiments were performed with a Roche LighCycler 2.0 (Roche, Germany).

Primer sequences were (5′ > 3′) Adiponectin-FW: GGC CGT GAT GGC AGA GAT, Adiponectin-REV: CTT CAG CCC CGG GTA CT; HPRT-FW: GAG ATG GGA GGC CAT CAC ATT GTA GCC CTC, HPRT-REV: CTC CAC CAA TTA CTT TTA TGT CCC CTG TTG ACT GGT C; CCL20-FW: TGG GAT CTC GTT GGA AAT AAC AC, CCL20-REV: GCA TTG ATG TCA CAG CCT TCA TTG GCC AG; IRAK1-FW: GGA GAC ATC AAG AGT TCC AAC GTC CTT CTG, IRAK1-REV: GTC TTT CAG ATA TTG GTC CTG GCA CCG T; IL-6-FW: TAC CCC CAG GAG AAG ATT CC, IL-6-REV: TTT TCT GCC AGT GCC TCT TT; IL-8-FW: CTG TGT GAA GGT GCA GTT TTG CC, IL-8-REV: CTT CTC CAC AAC CCT CTG CAC CC; MCP-1-FW: TCC CAA AGA AGC TGT GAT CTT CAA GAC C, MCP-1-REV: AGT GAG TGT TCA AGT CTT CGG AGT TTG G; SDHA-FW: CAT GCT GCC GTG TTC CGT GTG GG, SDHA-REV: GGA CAG GGT GTG CTT CTT CCA GTG CTC C; TRAF6-FW: ATC AAT AAG GGA TGC AGG TCA CAA ATG TCC A, TRAF6-REV: GAT GTC TCA GTT CCA TCT TGT GCA AAC AAC CT.

### Western Blot

Cells were washed with PBS and harvested in a lysis buffer consisting of 10 mM Tris-HCl, 150 mM NaCl, 2 mM EDTA, 1% TX-100, 10% glycerol, 1 mM DTT and cOmplete Protease Inhibitor Cocktail (Roche, Germany). Thirty μg protein lysates were separated by electrophoresis using 4–12% NuPAGE Novex Bis-Tris polyacrylamide gels (Life Technologies, Germany) and NuPAGE MES as running buffer. iBlot 7-Minute Blotting System from Life Technologies was used to achieve protein transfer onto nitrocellulose membranes. The membranes were blocked in 5% milk in TBST for one hour at room temperature. Incubations with primary antibodies were followed by incubations with the appropriate secondary antibodies conjugated with HRP and by detection using Amersham ECL Western Blotting Detection Reagents and Hyperfilms (GE Healthcare, Germany). Antibodies used in the study were α-tubulin (Calbiochem, Germany), β-actin, ERK 1/2 (Sigma Aldrich, Germany), AKT, IκBα, p38, pAKT (S473), pERK 1/2 (T202/Y204), pIκBα (S32/S36), pJNK (T183/Y185), pp38 (T180/Y182) (Cell Signaling, USA), JNK (BD Bioscience, USA), TRAF6 (Invitrogen, Germany), IRAK1, mouse IgG and rabbit IgG (Santa Cruz, USA).

### ELISA

To assess the secretion of IL-8 and MCP-1 by adipocytes, cell culture supernatants were collected, cleared by centrifugation and stored at −80 °C until analysis. For quantification of human IL-8 and MCP-1 the Human IL-8 ELISA Ready-SET-Go! (2^nd^ Generation) Kit and the Human MCP-1 ELISA Ready-SET-Go! Kit (2^nd^ Generation) were used (eBioscience, Germany). Assays were performed according to the manufacturer’s protocols.

### NF-κB electrophoretic mobility shift assay (EMSA)

Nuclear protein extraction and NF-κB EMSA were performed as described previously^28^. Briefly, T4 polynucleotide kinase (MBI Fermentas, Germany) was used to label NF-κB sense probe (5′-AGT TGA GGG GAC TTT CCC AGG C-3′) with γ-[32 P]-ATP (Perkin Elmer, USA). A 2-fold excess of unlabelled NF-κB antisense probe (5′-GCC TGG GAA AGT CCC CTC AAC T-3′) was added and annealed. The labelled NF-κB probe was purified with Micro Bio-Spin P30 columns (BioRad, Germany).

Five μg nuclear extract protein was incubated with 1 μg poly(dI:dC) (Sigma-Aldrich, Germany) and 10,000–12,000 cpm of labelled NF-κB probe for 30 minutes at room temperature. The formed complexes were separated on a 6% polyacrylamide gel which was then dried onto Whatman paper (GE Healthcare, Germany). To detect the radioactive signal Amersham Hyperfilm (GE Healthcare, Germany) was used.

### Statistics

GraphPad Prism (version 6.01c) software was used to perform statistical analysis. For comparison between two groups, the student’s t-test was used whereas for three or more groups with two independent variables, two-way ANOVA was used. Multiple comparisons were corrected with Bonferroni’s post-test. Statistical significance was assumed for p-values ≤ 0.05. If not stated otherwise data is given in mean ± SEM.

## Results

### miR-146a is up-regulated in adipocytes under inflammatory conditions

**Figure 1:**
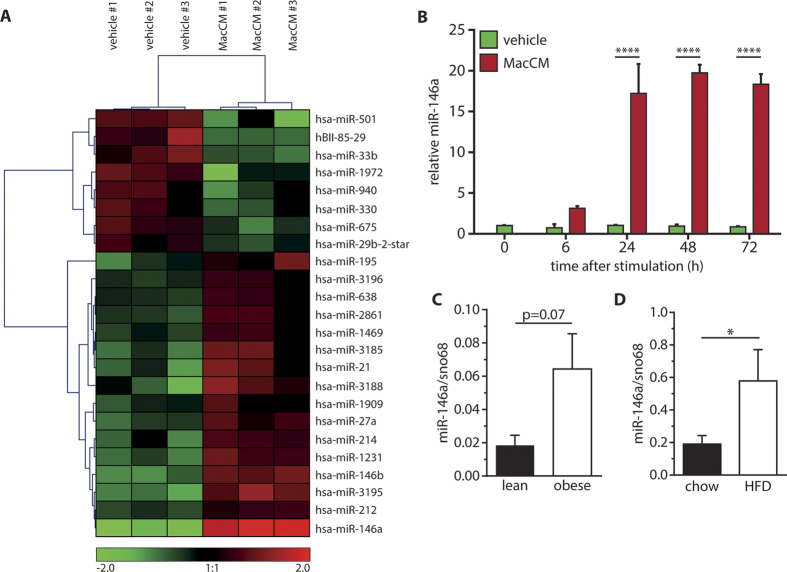
Differential miRNA expression in SGBS adipocytes under inflammatory conditions. **(A)** SGBS adipocytes were treated with 10% of human THP-1 macrophage-conditioned media (MacCM) or the corresponding vehicle control. Total RNA was isolated after 48 h. **(A)** The miRNA expression profile was assessed by an Affymetrix GeneChip miRNA Array 2.0. Results of three biological replicates are shown. **(B)** SGBS adipocytes were treated with 10% MacCM. Total RNA was harvested after 0, 6, 24, 48 and 72 h and reverse transcribed. The expression of miR-146a was assessed by qPCR. Data were analysed using the ΔΔCT method with sno68 as reference gene. The results are displayed as mean and SEM of three independent experiments. **(C)** miR-146a expression in subcutaneous adipose tissue of lean and obese females (n = 18 total) related to sno68. **(D)** miR-146a expression in subcutaneous adipose tissue from mice, which had been fed a high fat (n = 3) or chow diet (n = 5) for 8 weeks related to sno68. The results are displayed as mean and SEM. Statistics: **(B)** Two-way ANOVA, (**C** and **D**) paired t-test, *p < 0.05, ****p < 0.0001.

To study the influence of a pro-inflammatory stimulus on the miRNA expression profile of human SGBS adipocytes, we took advantage of a recently established *in vitro* model of adipose tissue inflammation^26^. SGBS adipocytes on day 14 of adipogenic differentiation were treated with 10% MacCM or the corresponding vehicle control. After 48 h, total RNA was isolated and an Affymetrix GeneChip miRNA Array 2.0 was used to investigate the miRNA expression profile. The cluster analysis revealed 24 miRNAs which were differentially regulated between MacCM- and vehicle-treated cells with the confidence level set at 0.05 (Fig. 1A). The miRNA which was the most up-regulated by MacCM treatment was miR-146a (12.15-fold). This up-regulation was also confirmed by qPCR analysis (Supplement Figure 1). To further support this finding, a time course analysis was performed (Fig. 1B). After 6 h of MacCM stimulation miR-146a levels were already increased. After 24 h, 48 h and 96 h miR-146a was significantly up-regulated 17.2-, 19.8- and 18.3-fold as shown by qPCR analysis.

### miR-146a is upregulated in adipose tissue in human and murine obesity

To establish a possible role of miR-146a in adipose tissue *in vivo*, we measured its expression in human and murine obesity. In a cohort of 18 females undergoing plastic surgery, the relative expression of miR-146a in subcutaneous adipose tissue tended to be higher in obese (n = 10; BMI: 33.55 ± 0.82 kg/m^2^) compared to lean (n = 8; BMI: 23.11 ± 0.33 kg/m^2^) subjects (mean obese: 0.064 *vs* mean lean: 0.018, p = 0.07) (Fig. 1C). Along the same line, the expression of miR-146a was significantly higher in subcutaneous adipose tissue from mice that had been fed a high fat diet for 8 weeks compared to mice on a chow diet (Fig. 1D). Body weights and fat pad weights are given in Supplementary Figure 2.

### miR-146a dampens the inflammatory response in human adipocytes

**Figure 2:**
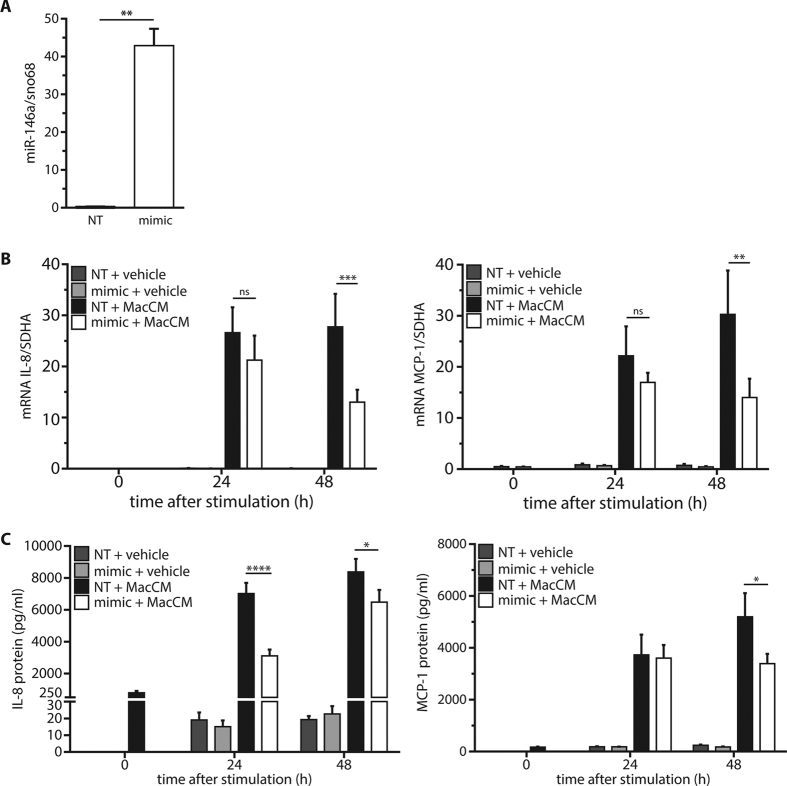
miR-146a down-regulates the inflammatory response in SGBS adipocytes. SGBS adipocytes were transfected with 20 nM miR-146a mimic or a non-targeting control (NT). **(A)** 48 h post transfection, the successful delivery of miR-146a was assessed by qPCR with sno68 as reference gene (2^−ΔCT^). The results are displayed as mean and SEM of four independent experiments. **(B)** 48 h post-transfection, cultures were stimulated with 10% MacCM or vehicle. RNA was isolated at 0, 24, and 48 h post-stimulation. IL-8 and MCP-1 mRNA expression were assessed by RT-qPCR with SDHA as reference gene. The results are displayed as mean and SEM of four independent experiments. **(C)** IL-8 and MCP-1 were measured in media supernatants by ELISA. The results are displayed as mean and SEM of three independent experiments performed in duplicates. Statistics: **(A)** paired t-test, (**B** and **C**) two-way ANOVA, ns = not significant, *p < 0.05, **p < 0.01, ***p < 0.001, ****p < 0.0001.

**Figure 3:**
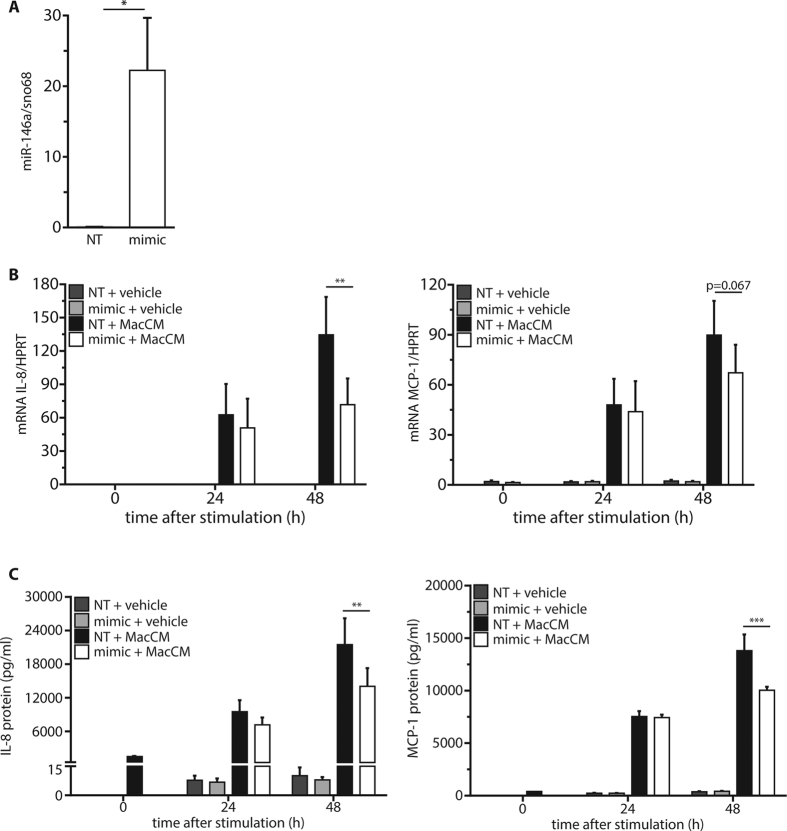
miR-146a reduces the inflammatory response in human primary adipocytes. Stromal-vascular cells isolated from WAT of three females were subjected to adipogenic differentiation. On day 14, the cells were transfected with 20 nM miR-146a mimic or a non-targeting control (NT). **(A)** 48 h post transfection, the successful delivery of miR-146a was assessed by qPCR with sno68 as reference gene (2^−ΔCT^). The results are displayed as mean and SEM of all three subjects. **(B)** 48 h post-transfection, cultures were stimulated with 10% MacCM or vehicle control. RNA was isolated at 0, 24, and 48 h post-stimulation. IL-8 and MCP-1 mRNA expression were assessed by RT-qPCR with SDHA as reference gene. The results are displayed as mean and SEM of all three subjects. **(C)** IL-8 and MCP-1 were measured in media supernatants by ELISA. The results are displayed as mean and SEM of all three subjects with measurements performed in duplicates. Statistics: **(A)** paired t-test, (**B** and **C**) two-way ANOVA, *p < 0.05, **p < 0.01, ***p < 0.001.

To investigate the role of miR-146a on the inflammatory response in human adipocytes, a gain-of-function approach was chosen. SGBS adipocytes were transfected with a synthetic miR-146a mimic. Forty-eight hours post-transfection, the intracellular levels of miR-146a were significantly increased by ~300-fold compared to cells transfected with a control oligonucleotide (Fig. 2A). Upon MacCM stimulation, control cells showed a ~1500-fold increase in mRNA expression of the pro-inflammatory interleukin IL-8 and a ~65-fold increase of the cytokine MCP-1 (Fig. 2B). Adipocytes transfected with miR-146a showed significantly reduced levels of IL-8 (~53% reduction) and MCP-1 (~54% reduction) mRNA after 48 h of stimulation. The reduced production of IL-8 and MCP-1 was confirmed on the protein level by measuring their accumulation in media supernatants by ELISA (Fig. 2C). For example, the IL-8 secretion was reduced by miR-146a transfection by 45% after 24 h and by 23% after 48 h and MCP-1 secretion was reduced by 35% after 48 h. The mRNA expression of other pro-inflammatory mediators produced by adipocytes like IL-6 and CCL20 was also reduced by ~40% and ~50% after 48 h of MacCM treatment (Supplement Figure 3A). However, adiponectin which is a typical anti-inflammatory adipokine was not affected by mimic transfection (Supplement Figure 3B). To exclude that the effect of miR-146a is restricted to SGBS cells, the same set of experiments was performed in *ex vivo* differentiated primary human adipocytes obtained from subcutaneous adipose tissue of three different female donors (age: 33.3 ± 8.25 years; BMI: 24.3 ± 1.39 kg/m^2^). Results with human primary cells were comparable to those with SGBS cells (Fig. 3A–C).

### IRAK1 and TRAF6 are targets of miR-146a in human adipocytes

There is accumulating evidence in the literature that miR-146 plays a role in the regulation of inflammatory responses. It is described as a negative regulator of signaling pathways leading to NF-κB activation. Well-known targets of miR-146a include TNF receptor-associated factor 6 (TRAF6) and IL-1 receptor-associated kinase 1 (IRAK1), which act downstream of the IL-1 receptor and toll-like receptors^29^,^30^.

**Figure 4:**
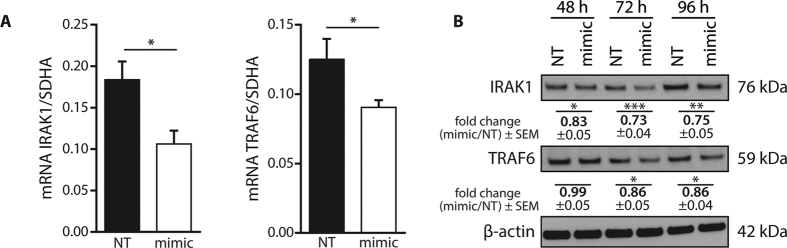
IRAK1 and TRAF6 are targets of miR-146a in SGBS adipocytes. SGBS adipocytes were transfected with 20 nM miR-146a mimic or a non-targeting control (NT). **(A)** Total RNA was isolated after 48 h. IRAK1 and TRAF6 mRNA levels were assessed by qPCR with SDHA as reference gene (2^−ΔCT^). Data is shown as mean and SEM of four independent experiments. **(B)** Protein was extracted after 48, 72 and 96 h. Expression of IRAK1 and TRAF6 was assessed by Western blot with β-actin as loading control. One representative out of at least four independently performed experiments is shown. Densitometric fold changes are displayed as mean and SEM of all experiments. Statistics: paired t-test, *p < 0.05, **p < 0.01, ***p < 0.001.

The delivery of miR-146a mimic into mature SGBS adipocytes (Fig. 2A) resulted in a decrease of 43% for IRAK1 and 28% for TRAF6 mRNA expression after 48 h (Fig. 4A). On the protein level, IRAK1 was down-regulated by up to 27%, while TRAF6 showed a maximum down-regulation by 14% compared to control cells (Fig. 4B).

### miR-146a reduces the pro-inflammatory signaling in human adipocytes

**Figure 5:**
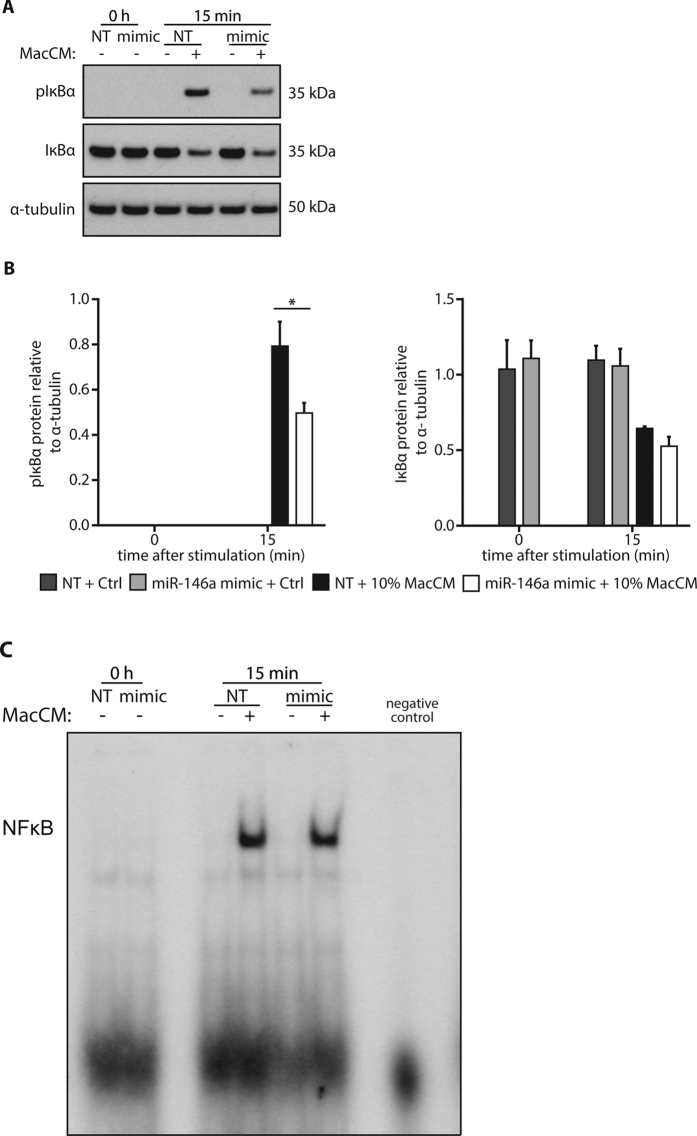
Effects of miR-146a on the NF-κB pathway. **(A)** SGBS adipocytes were transfected with 20 nM miR-146a mimic or a non-targeting control (NT). 48 h post transfection, cells were stimulated with 10% MacCM or vehicle. Protein was extracted at 0 and 15 min. Western blots using specific antibodies against pIκBα and IκBα were performed with α-tubulin as loading control. One representative out of three independently performed experiments is shown. **(B)** Densitometric changes are displayed as mean and SEM of all experiments. Statistics: paired t-test, *p < 0.05. (**C**) Nuclear proteins were isolated and subjected to EMSA to assess the transcriptional activity of NF-κB. One representative of three independently performed experiments is shown.

**Figure 6:**
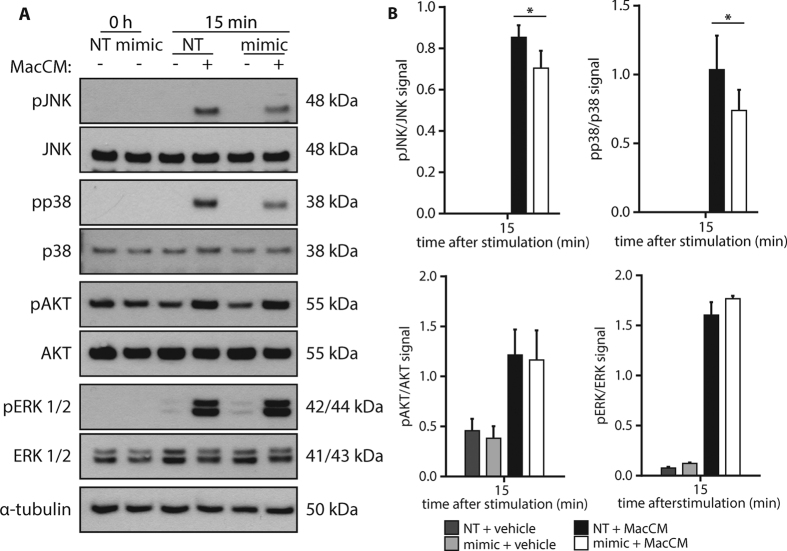
miR-146a mimic reduces MacCM-induced phosphorylation of JNK and p38. **(A)** SGBS adipocytes were transfected with 20 nM miR-146a mimic or non-targeting control (NT). 48 h post transfection, cells were stimulated with 10% MacCM or vehicle. Protein was extracted at 0 and 15 min. Western blots using specific antibodies again pJNK/JNK, pp38/p38, pAkt/Akt and pERK1/2/ERK were performed with α-tubulin as loading control. One representative out of four independently performed experiments is shown. **(B)** Densitometric changes are displayed as mean and SEM of all experiments. Statistics: paired t-test, *p < 0.05.

In the next step, we wanted to know if the miR-146a-mediated down-regulation of IRAK1 and TRAF6 has a functional consequence and impacts on MacCM-induced signal transduction. Therefore, mimic or control-transfected adipocytes were stimulated with 10% MacCM for 15 min and the activation of several intracellular signaling pathways was analyzed. Our prime candidate for those studies was the NF-κB pathway. As a surrogate of its activation, phosphorylated and total IκBa was measured by Western blot analysis. As expected, a robust phosphorylation of IkBa was detected upon MacCM stimulation in control cells, which resulted in a clear down-regulation of total IκBa protein (Fig. 5A). Interestingly, delivery of miR-146a mimic resulted in a 35% weaker phosphorylation of IκBa, yet the level of protein degradation was comparable to control cells (Fig. 5B), which might argue that the actual transmission of the signal is comparable. We therefore performed an electrophoretic mobility shift assay (EMSA), one of the gold standards of NF-κB activation. In line with our assumption, MacCM triggered the transcriptional activity of NF-κB to a comparable extent in miR-146a and control transfected cells (Fig. 5C). From this set of data, we conclude that NF-κB is not involved in mediating the miR-146a effect on IL-8 and MCP-1 production. We then studied the activation of other protein kinase pathways possibly involved in the regulation of the inflammatory response. Western blot analyses demonstrated that AKT, ERK1/2, p38 and JNK expression levels were not altered by miR-146a transfection (Fig. 6A). All four kinases were phosphorylated upon MacCM stimulation. While miR-146a delivery resulted in a significant down-regulation of phospho-JNK by ~20% and phospho-p38 by ~30%, the activation of AKT and ERK1/2 was not altered (Fig. 6B). From these experiments we conclude that JNK and p38 are involved in mediating the observed miR-146a effects.

## Discussion

Obesity is associated with chronic low-grade inflammation in adipose tissue. This inflammatory state alters adipocyte function and is positively correlated with a decreased metabolic control promoting obesity-related diseases such as type 2 diabetes mellitus^31^. miRNAs are modulators of the immune response and especially miR-146a is known to be a fine tuner of the inflammatory response, which possibly prevents uncontrolled inflammation^32^. We identified miR-146a by an Affymetrix miRNA array as a miRNA that is strongly upregulated in adipocytes under inflammatory conditions. Based on this, gain-of-function studies were performed to investigate the role of miR-146a in human adipocyte inflammation.

### microRNA-146a is elevated under inflammatory conditions

To mimic WAT inflammation, adipocytes were treated with macrophage conditioned medium (MacCM). This is a well-established model system in our lab. MacCM is composed of different cytokines and chemokines including TNF-α, IL-6 and IL-1β^26^. It exerts pleiotropic effects, but mimics the inflammatory micro-environment found in WAT *in vivo*^26^. Here we show that the stimulation with MacCM induces an inflammatory response and results in a significant upregulation of miR-146a. These findings closely match results gained in other cell types. For example, miR-146a levels are elevated in chronically inflamed tissues of psoriasis and rheumatoid arthritis patients^33^,^34^ and in *in vitro* studies in several human cell types, including monocytes^21^, astrocytes^35^, lung epithelial cells^20^ and umbilical vein endothelial cells^36^. With our study we now add human adipocytes to the list of cells, which display an miR-146a upregulation under inflammatory conditions. This is also reflected on the whole tissue level, as miR-146a was higher expressed in obese compared to lean WAT in both mice and humans.

### microRNA-146a reduces the inflammatory response in adipocytes

The impact of miR-146a on the inflammatory response in adipocytes was assessed by analysing the expression and secretion of pro-inflammatory adipokines. Transfection with miR-146a mimic reduced the MacCM-induced upregulation of IL-6, IL-8, MCP-1 and CCL-20 on mRNA level. Even more importantly, the mimic also reduced the accumulation of IL-8 and MCP-1 in the media supernatants. Similar results were already reported in other cell types such as human keratinocytes^19^, human alveolar epithelial cells^20^ and human astrocytes^35^. To induce an inflammatory response, Meisgen *et al*. used the glucan Zymosan, an activator of TLR2, while Perry *et al*. and Iyer *et al*. stimulated cells with IL-1β. All three studies showed a reduction of pro-inflammatory molecules due to miR-146a mimic transfection^19^,^20^,^35^, which is in line with our results. We conclude from our data that miR-146a is a crucial regulator of inflammation and dampens the inflammatory response in human adipocytes.

We want to point out that it is possible that the regulating effects of miR-146a in adipocytes go beyond a single cell. Since adipocytes communicate with other cells *via* microvesicles in a paracrine manner^37^ it might be possible that elevated miR-146a secretion from inflamed adipocytes suppresses pro-inflammatory signalling in surrounding adipocytes as well as in other cell types within the adipose tissue. This assumption is substantiated by the observation, that 140 different miRNAs were found in exosomes from a mouse adipocyte cell line^37^. Considering that miRNAs are not limited to a single cell but might influence processes in surrounding cells, it is conceivable that adipocyte-derived miR-146a also has an influence on macrophages within the adipose tissue. The phenotype switch of anti-inflammatory M2-polarized to pro-inflammatory M1-polarized macrophages in obese adipose tissue plays a crucial role in adipose tissue inflammation^38^ and therefore adipocyte-secreted miR-146a possibly protects adipose tissue from an overwhelming inflammatory response.

miRNAs released from cells are found in the blood stream and can act systemically^39^. A clinical study compared serum levels of miR-146a and clinical parameters of inflammation in patients with type 2 diabetes with those in a non-diabetic control group^22^. This study reported a significant reduction of miR-146a and increased pro-inflammatory IL-8 in serum of type 2 diabetes patients compared to the control group, revealing a direct link between miR-146a serum levels, inflammation and a failure in glucose homeostasis^22^.

### microRNA-146a mediates the inflammatory response *via* TRAF6 and IRAK1

As a potential molecular mode of action, we identified IRAK1 and TRAF6 as targets of miR-146a. Studies comparable to our work using miRNA mimics were performed in human keratinocytes^19^, human breast cancer cells^40^ and alveolar epithelial cells^20^, all showing that IRAK1 and TRAF6 are targets of miR-146a. Furthermore, a miR-146a knock-out mouse model confirmed IRAK1 and TRAF6 as targets of miR-146a by profiling primary macrophages derived from wild-type and knock-out animals^16^. Therefore, it is not surprising that IRAK1 and TRAF6 are also targeted and down-regulated by miR-146a in human adipocytes. Furthermore, we showed that transfection with miR-146a mimic led to alterations in cellular signalling pathways downstream of TRAF6 and IRAK1, *e.g.* a reduced phosphorylation of IκBα, the inhibitor of NF-κB. This result fits to the findings of Boldin *et al*. who reported a dysregulation of NF-κB signalling in miR-146a knockout-mice and of Bhaumik *et al*., who showed that miR-146a supresses NF-κB signalling in human breast cancer cells^16^,^40^. In contrast to the phosphorylation of IκBα, its degradation, which is the crucial event for NF-κB activation, was not altered due to miR-146a mimic transfection. In consequence we found also no difference between control and miR-146a transfected cells in the transcriptional activity of NF-κB which was assessed by EMSA. This might indicate that miR-146a does not influence NF-κB signalling in adipocytes.

Besides NF-κB, IL-8 expression and the expression of other pro-inflammatory adipokines is additionally regulated by p38, JNK and ERK1/2^41^,^42^. Therefore, the activation of these kinases, which occurs by phosphorylation, was assessed. As with IκBα, the MacCM-induced phosphorylation of JNK and p38 was significantly reduced in cells transfected with miR-146a mimic. ERK1/2 phosphorylation was not affected. Reduced JNK, and p38 activation by miR-146a mimic transfection was already reported for macrophages by Yang *et al*.^43^.

In summary we identified miR-146a in human adipocytes as a fine tuner of the inflammatory response acting as a negative feedback regulator of WAT inflammation through downregulation of the pro-inflammatory IRAK1/TRAF6 pathways. Since miRNA therapeutics evolved from bench to bedside^44^ it might be possible to use miR-146a mimics as a tool to maintain WAT homeostasis by controlling the inflammatory processes within the WAT.

## Additional Information

**How to cite this article**: Roos, J. *et al*. miR-146a-mediated suppression of the inflammatory response in human adipocytes. *Sci. Rep.* **6**, 38339; doi: 10.1038/srep38339 (2016).

**Publisher's note:** Springer Nature remains neutral with regard to jurisdictional claims in published maps and institutional affiliations.

## Supplementary Material

Supplementary Information
