# Supplementary material for: miR-146a-mediated suppression of the inflammatory response in human adipocytes
Source: Sci Rep. 2016 Dec 6;6:38339. doi: 10.1038/srep38339 (PMC5138634; doi:10.1038/srep38339)
Supplement: Supplementary Information [file srep38339-s1.pdf]

Supplementary Appendix

miR-146a-mediated suppression of the inflammatory response in human adipocytes

Authors:

Julian Roos[1], Eveliina Enlund[1], Jan-Bernd Funcke[1], Daniel Tews[1], Karlheinz Holzmann[3], Klaus-Michael Debatin[2], Martin Wabitsch[1], and Pamela Fischer-Posovszky[1]

Affiliations: [1] Division of Pediatric Endocrinology and Diabetes, Department of Pediatric and Adolescent Medicine, University Medical Center Ulm, Ulm, Germany; [2] Department of Pediatric and Adolescent Medicine, University Medical Center Ulm, Ulm, Germany; [3] Core Facility Genomics, Ulm University, Ulm, Germany

Correspondence and reprint requests:

Dr. Pamela Fischer-Posovszky

Division of Pediatric Endocrinology and Diabetes

Department of Pediatrics and Adolescent Medicine

University Medical Center Ulm

Eythstr. 24, 89075 Ulm, Germany

tel.: +49 731 500 57401

fax.: +49 731 500 57407

email: pamela.fischer@uniklinik-ulm.de

Supplement Figure 1

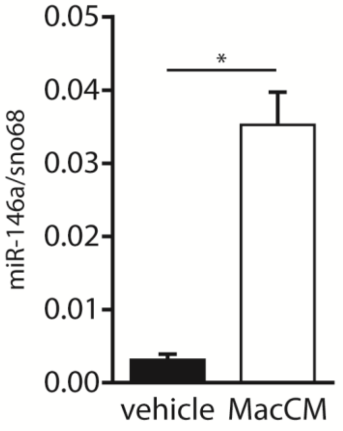

Supplement Figure 1: Array validation by qPCR

(A) SGBS adipocytes were treated with 10% of human THP-1 macrophage-conditioned media (MacCM) or the corresponding vehicle control. Total RNA was isolated after 48 h and reverse transcribed. The expression of miR-146a was assessed by qPCR with sno68 as reference gene. The results are displayed as mean and SEM of three independent experiments. Statistics: paired t-test, *p<0.05

Supplement Figure 2

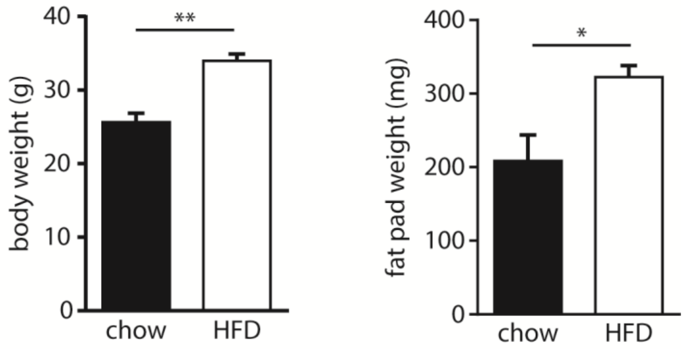

Supplement Figure 2: Body weight and fad pad weight of mice on chow and high fat diet

Body weight **(A)** and inguinal fat pad weight **(B)** of mice fed either a high fat (n=3) or chow diet (n=5) for 8 weeks. Data are displayed as mean and SEM. Statistics: paired t-test, * p< 0.05, ** p< 0.01

Supplement Figure 3

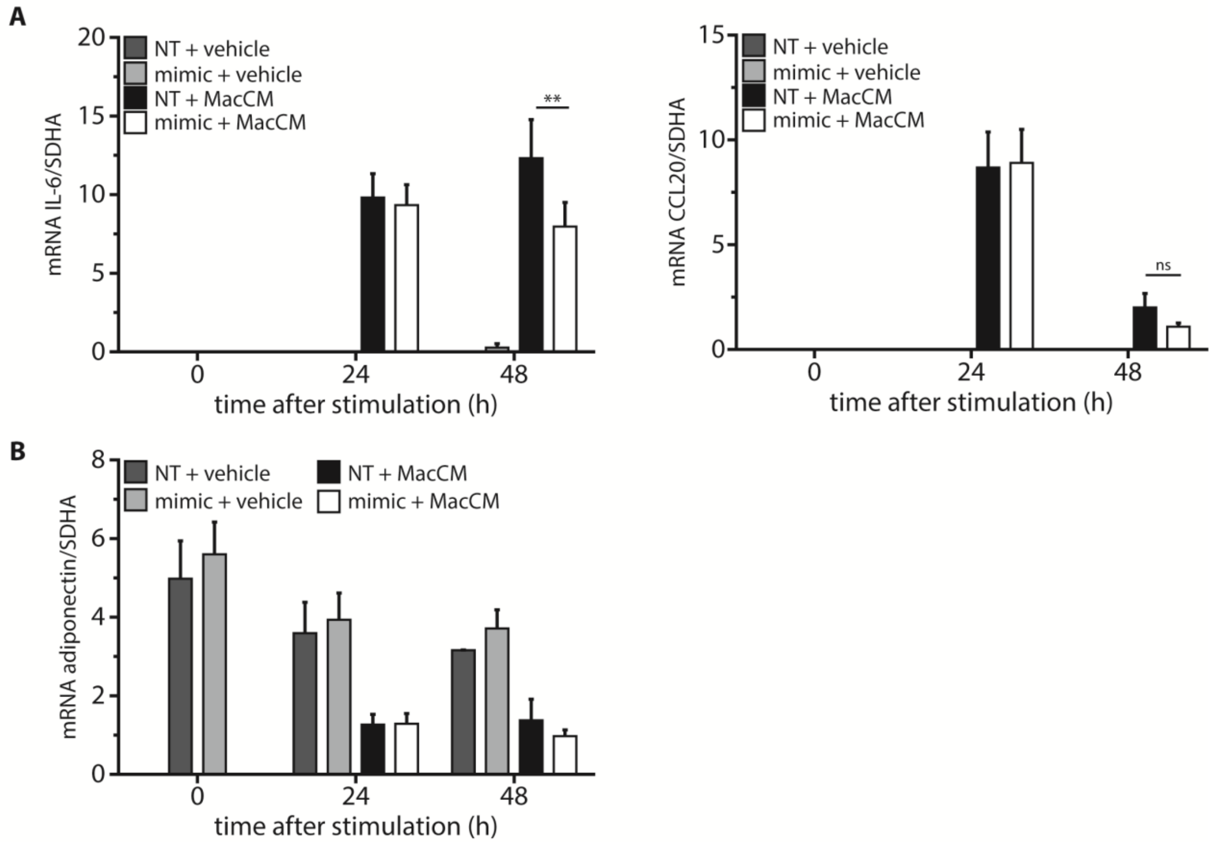

Supplement Figure 3: miR-146a down-regulates IL-6 and CCL20 expression

SGBS adipocytes were transfected with 20 nM miR-146a mimic or a non-targeting control siRNA (NT). **(A** & **B)** 48 h post-transfection, cultures were stimulated with 10% MacCM or vehicle control. RNA was isolated at 0, 24, and 48 h post-stimulation. IL-6, CCL20 and adiponection mRNA expression was assessed by RT-qPCR with SDHA as reference gene. The results are displayed as mean and SEM of four independent experiments. Statistics: two-way ANOVA, ns = not significant, ** $p < 0.01$
